# Interface Engineered Binary Platinum Free Alloy-based Counter Electrodes with Improved Performance in Dye-Sensitized Solar Cells

**DOI:** 10.1038/s41598-020-64965-7

**Published:** 2020-06-08

**Authors:** Wen-Wu Liu, Wei Jiang, Yu-Cheng Liu, Wen-Jun Niu, Mao-Cheng Liu, Ling-Bin Kong, Ling Lee, Zhiming M. Wang, Yu-Lun Chueh

**Affiliations:** 1 State Key Laboratory of Advanced Processing and Recycling of Nonferrous Metals, Lanzhou University of Technology, Lanzhou, 730050 PR China; 2 College of Materials Science and Engineering, Lanzhou University of Technology, Lanzhou, 730050 PR China; 3 Department of Materials Science and Engineering, National Tsing Hua University, Hsinchu, 30013 Taiwan; 4 Frontier Research Center on Fundamental and Applied Sciences of Matters, National Tsing Hua University, Hsinchu, 30013 Taiwan; 5 Department of Physics, National Sun Yat-Sen University, Kaohsiung, 80424 Taiwan; 6 Institute of Fundamental and Frontier Sciences, University of Electronic Science and Technology of China, Chengdu, P. R. China

**Keywords:** Energy science and technology, Engineering, Physics

## Abstract

The high cost and platinum dissolution issues of counter electrodes (CEs) are two core problems for the development of dye-sensitized solar cells (DSSCs). In this work, different CEs based on binary alloy Ru_81.09_Co_18.91_, Ru_80.55_Se_19.45_ and Co_20.85_Se_79.15_ nanostructures for DSSCs were successfully synthesized and investigated by a facile and environmentally friendly approach. Here, we found that the Co_20.85_Se_79.15_ alloy CE-based device yields the higher photoelectric conversion efficiency of 7.08% compared with that (5.80%) of the DSSC using a pure Pt CE because of the larger number of active sites with improved charge transferability and reduced interface resistance by matching work function with the I_3_^‒^/I^‒^ redox electrolyte. The inexpensive synthesis method, cost-effectiveness and superior catalytic activity of the Co_20.85_Se_79.15_ alloy may open up a new avenue for the development of CEs for DSSCs in the near future.

## Introduction

Advanced electrocatalysts play a crucial role in fuel cells, air batteries and dye-sensitized solar cells (DSSCs)^1–3^. Platinum (Pt) metal is commonly considered a good electrocatalyst due to its superior electrocatalytic activity in the I_3_^‒^ reduction reaction at the electrolyte/counter electrode (CE) interface, good electrical conductivity, and high sunlight reflection ability in DSSCs^4^. However, the Pt metal suffers from drawbacks, such as expensive cost and unstable stability because of a dissolution reaction exposed to the I_3_^‒^/I^‒^ redox electrolyte. Particularly, the dissolution loss of the Pt metal may reduce the long-term stability of the photovoltaic efficiency of DSSCs. It is very important that developing a novel type of electrocatalyst has been one of the key objectives in studies focused on DSSCs. However, the most current research aimed at enhancing the electrocatalytic performance is still limited to the partial substitution of Pt with conductive polymers or carbon-based materials, or to the complete substitution of Pt with metal carbides, oxides, sulfides, and selenides^5–7^. The structure of these CEs would also be destroyed by the I_3_^‒^/I^‒^ based electrolyte under long-term electrochemical cycling^8^.

Consequently, metal alloy-based electrocatalysts have attracted considerable attention due to their outstanding properties. Generally, these alloys involve the combination of Pt meal with transition metal elements denoted as PtM_*x*_ compounds (M = Fe, Co, Ni, Pd, Ru, Cu, etc.)^9–11^. Work function calculation and experimental analyses indicate that binary PtM_*x*_ alloys have better electrocatalytic properties toward I_3_^‒^ reduction^11–13^. It has been reported that the decreased resistance at the PtM_*x*_/electrolyte interface and the competitive dissolution reaction between Pt and the transition metal effectively contribute to the enhanced electrocatalytic ability and stability of CEs^14,15^. Different lattice parameters in the structure of PtM_*x*_ alloys would result in ligand and strain effects^16^, thereby generating very large numbers of active sites for the reduction reaction of I_3_^‒^ ions. Nevertheless, the work functions of some bimetallic PtM_*x*_ alloys, such as PtCo, PtRu, or PtPd do not match the potential of the I_3_^‒^/I^‒^- based electrolyte, leading to unsatisfactory electrocatalytic activity and electron transport^17–19^. Meanwhile, the high cost of Pt metal is still a crucial obstacle for the further development of DSSCs.

In recent years, perovskite solar cells have attracted considerable attention worldwide with a corresponding gradual decrease in the number of studies focused on bimetallic Pt-free alloy electrocatalysts for DSSCs. From this perspective, the development of Pt-free polymetallic alloys has become a very important subject in the electrocatalysis field. Yin *et al*. synthesized a novel N-doped-carbon coated CoSe_2_ on a 3D carbon cloth as a photocathode for DSSCs, which exhibited a good photoelectric efficiency (8.40%) and cycle stability^20^. A carbon shell coated CoSe_2_ nanoparticles catalyst-based DSSC was also reported, which gives the good conversion efficiency of 7.54%^21^. Wang *et al*. reported a Co_0.85_Se and Ni_0.85_Se CE -based DSSCs with high efficiency of 9.40 and 8.32%, respectively, which were synthesized by *in situ* growth using a hydrothermal method^22^. These results indicate that transition metal selenides have superior catalytic activities than the pristine Pt CE for DSSCs. In this case, for the purpose of reducing the fabrication cost and improving their electrocatalytic activity and stability in DSSCs, the Pt-free bimetallic alloys including RuCo, RuSe and CoSe -based CEs were synthesized by a simple electrodeposition approach. The exact compositions of Ru_81.09_Co_18.91_, Ru_80.55_Se_19.45_ and Co_20.85_Se_79.15_ can be confirmed by Energy Dispersive Spectrometer (EDS) and X-ray photoelectron spectroscopy (XPS) characterization. As a result, the power conversion efficiency of Co_20.85_Se_79.15_ CE-based DSSCs reached 7.08%, compared with the 5.80% value of pure Pt CE-based DSSCs. The improved catalytic performance can be attributed to the matching work function, a large number of active sites and reduced interface resistance.

## Results and discussion

**Figure 1:**
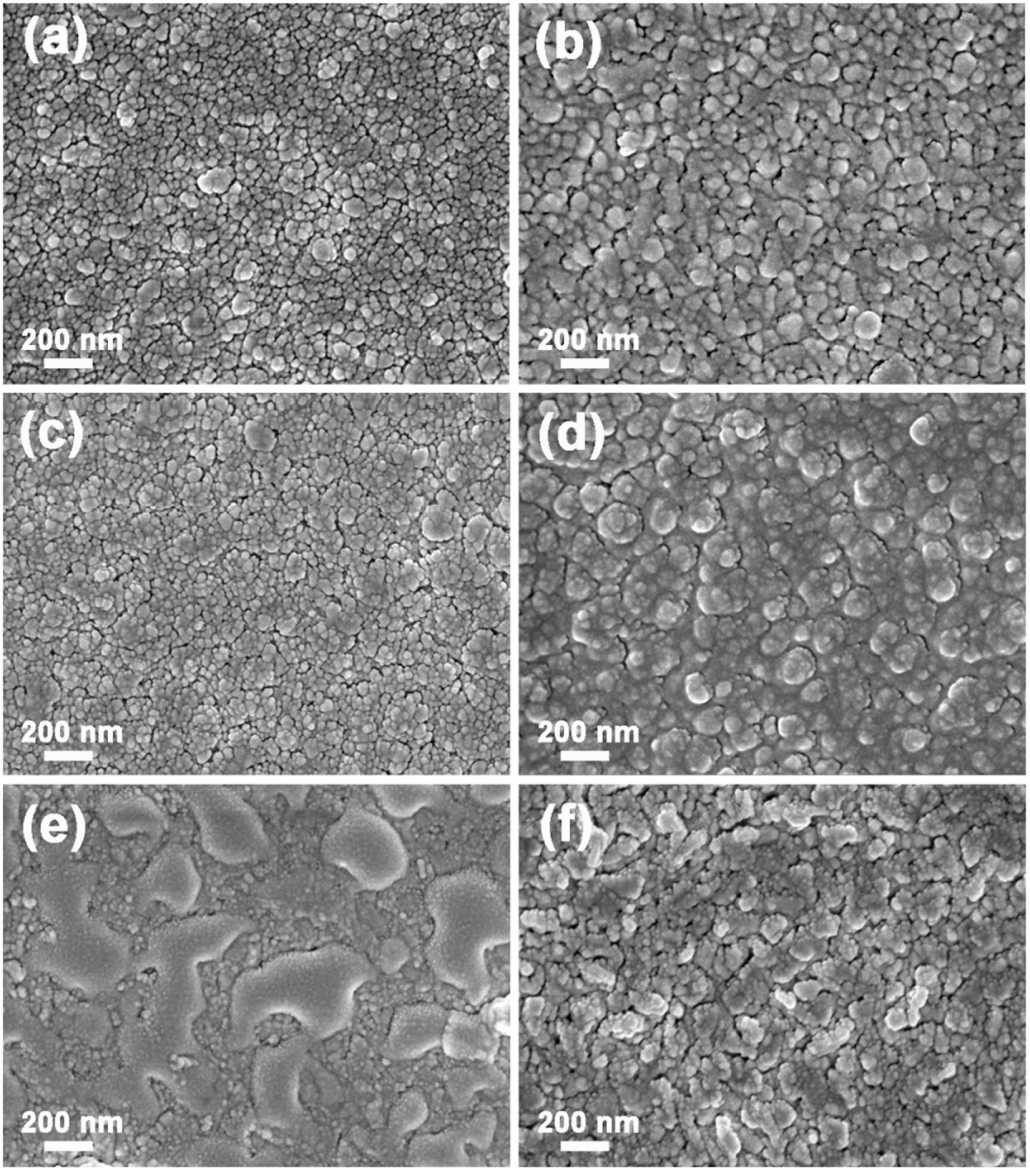
Top-view FESEM images of different alloys and pristine metal CEs at high magnification. (**a**) Ru_81.09_Co_18.91_, (**b**) Ru_80.55_Se_19.45_, (**c**) Co_20.85_Se_79.15_, (**d**) Ru, (**e**) Se, (**f**) Co.

High-magnification FESEM images of various alloy CEs electrodeposited on the surface of the FTO glass are shown in Fig. 1(a–f). The Ru_81.09_Co_18.91_, Ru_80.55_Se_19.45_, and Co_20.85_Se_79.15_ alloy CEs exhibited uniformly distributed surface morphologies compared to those of pure metal (Ru, Se, and Co) CEs. The alloy CEs showed a smaller average particle diameter and an almost homogeneous distribution (Fig. 1(a–c)), whereas a larger grain size and non-uniform surface appearance were observed for the pure metal CEs (Fig. 1(d–f)). XRD results of Co_20.85_Se_79.15_ alloy was characterized as shown in Figure S1, the peaks at 37.62°, 51.57° match well with (211) and (311) crystalline faces of CoSe_2_ (JCPDS PDF#09-0234), respectively. The peak at 51.52° can be well indexed to (200) face of Co (JCPDS PDF#15-0806). The peak at 33.33°, 61.57° can be well indexed to (101) and (103) face of Se (JCPDS PDF#27-0601). In order to confirm the composition of the metal alloys, the CEs were analyzed by the EDS method as shown in Figure S2 and the corresponding quantitatively compositional results were shown in Table 1, for which the atomic molar ratios of the alloys were found to be 1:0.4, 1:0.3, and 1.2:3.4, respectively. Therefore, the chemical compositions of the RuCo, RuSe and CoSe alloy CEs could be expressed as Ru_81.09_Co_18.91_, Ru_80.55_Se_19.45_ and Co_20.85_Se_79.15_, respectively.

**Table 1:** The quantitative EDS results of corresponding binary alloys CEs.

RuCo		RuSe		CoSe	
Element	Atomic ratio	Element	Atomic ratio	Element	Atomic ratio
Ru	1.0	Ru	1.0	Co	1.2
Co	0.4	Se	0.3	Se	3.4

**Figure 2:**
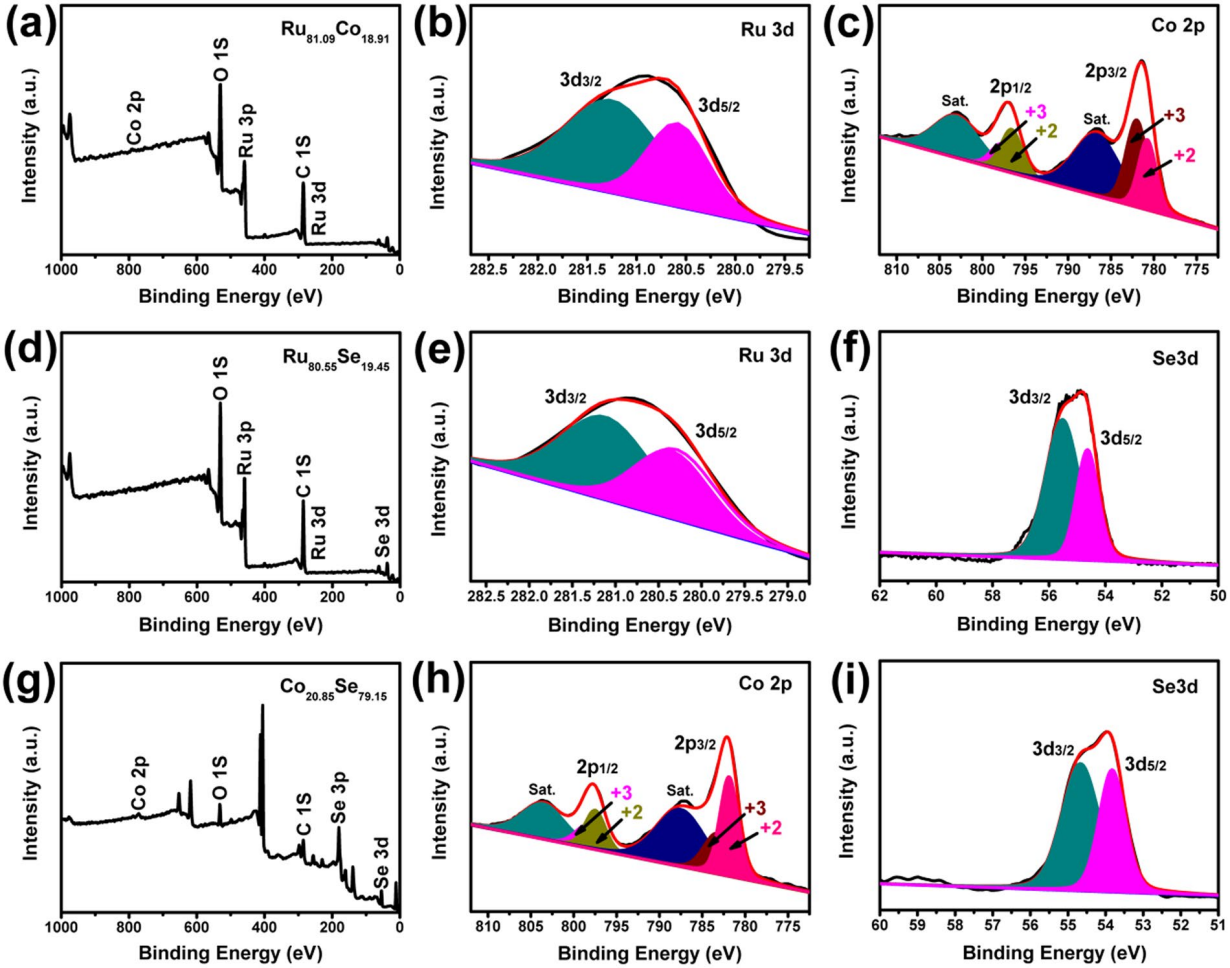
XPS spectra of Pt-free alloys (**a-c**) Ru_81.09_Co_18.91_, (**d-f**) Ru_80.55_Se_19.45_, (**g-i**) Co_20.85_Se_79.15_ CEs.

The X-ray photoelectron spectroscopy (XPS) spectra were also utilized to characterize the formation of different alloys as shown in Fig. 2. From Fig. 2(a,d,g), the Ru and Co, Ru and Se, Co and Se elements can be identified to be Ru_81.09_Co_18.91_, Ru_80.55_Se_19.45_ and Co_20.85_Se_79.15_, respectively. As can be shown in Fig. 2(b), the two peaks located at 280.5 and 281.2 eV were attributed to the bonding results of Ru-O and Ru-Ru. The Co 2p spectrum in Fig. 2(c) can be distributed into shakeup satellites (Sat.) and spin-orbit doublets. The first double peak at 780.7 and 796.6 eV, and the second speaks at 782.0 and 797.8 eV belong to Co^2+^ and Co^3+^, respectively^21–23^. The shakeup double peaks indicated the formation of the Co-Co bond^21–23^. Fig. 2(e) exhibits the Ru 3d spectrum of Ru_80.55_Se_19.45_ alloy while two peaks located at 280.3 and 281.1 eV were assigned to the bonding results of Ru-Se and Ru-Ru. The doublet peaks at 54.5 and 55.5 eV in Fig. 2(f) can be ascribed to the bonding results of Ru-Se and Se-Se^21,24^. Fig. 2(h) shows the Co 2p spectrum of Co_20.85_Se_79.15_ alloy. Similarly, the first doublet peaks at 780.9 and 796.5 eV, and the second speaks at 783.2 and 798.6 eV suggest the bonding results of Se-Co-Se and Co-O, respectively^21–23,25^. From Fig. 2(i), the two peaks at 53.8 and 54.6 eV were related to the bonding results of Co-Se and Se-Se, respectively^21,24^. The results indicated that the Co_20.85_Se_79.15_ alloy can be composed of CoSe_2_, Co_2_O_3_, Co and Se. It is believed that the alloy CEs can provide a higher number of active sites for the adsorption of I_3_^‒^ ions and their reduction reaction (I_3_^‒^ + 2e^‒^ = 3I^‒^), thereby accelerating the electrocatalytic reaction on the electrolyte/CEs interface as well as the transport of electrons. The smaller crystalline size of the alloy CEs may be explained in terms of the crystal growth occurring during the electrodeposition process. The multiple crystal nuclei on the alloy surface would contribute to the formation of smaller nanocrystals^26^.

**Figure 3:**
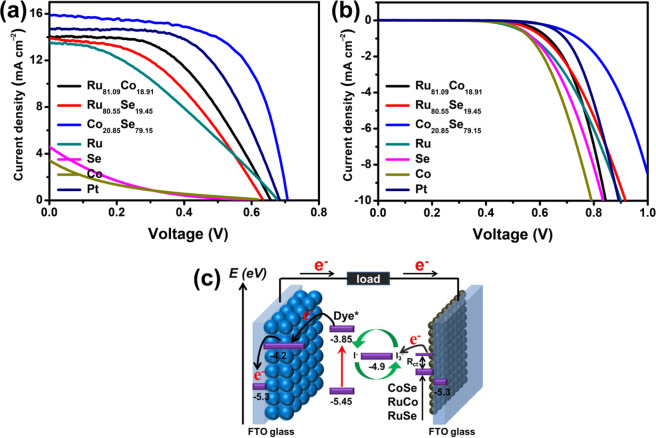
Photocurrent density-voltage curves of various CEs based DSSCs (**a**) with sunlight and (**b**) at dark condition, (**c**) device configuration of the alloy CEs supported DSSC.

Figure 3(a,b) display the photocurrent density-voltage plots of different CE-based DSSCs with and without sunlight illumination, respectively. Figure 3(c) shows the corresponding device configuration of the binary alloy CEs in DSSCs. The photoelectric parameters of the DSSCs are also shown in Table 2. Clearly, the transition metal alloys (Ru_81.09_Co_18.91_, Ru_80.55_Se_19.45_ and Co_20.85_Se_79.15_) considerably enhance the photoelectric conversion efficiency (PCE) of the DSSCs compared with that of pure metals (Ru, Se and Co). Improved PCEs of 4.57, 3.82 and 7.08% were achieved for the Ru_81.09_Co_18.91_, Ru_80.55_Se_19.45_ and Co_20.85_Se_79.15_ alloy CE-based DSSCs, respectively. In particular, the optimal DSSC based on Co_20.85_Se_79.15_ CE yielded a significantly improved photovoltaic performance (*J*_sc_ = 15.91 mA cm^‒2^, *V*_oc_ = 0.71, *FF* = 0.62 and *PCE* = 7.08%) compared with those of the Pt-based DSSCs (*J*_sc_ = 14.72 mA cm^‒2^, *V*_oc_ = 0.69, *FF* = 0.57 and *PCE* = 5.80%). Figure S3 exhibits the box charts, showing the statistical distribution of *J*_sc_, *V*_oc_, *FF* and *PCE* of the pristine Pt and Co_20.85_Se_79.15_ CE based DSSCs, respectively. The average *FF* (0.59) of Co_20.85_Se_79.15_ CE based devices is larger than that (0.55) of the pristine Pt-based DSSCs (Figure S3-c). Meanwhile, the average *PCE* of Co_20.85_Se_79.15_ based DSSCs is enhanced from 5.66% for the pristine Pt-based counterparts to 6.85% (Figure S3-d) as a result of the increased *J*_sc_ and *FF*. The improved performance of the DSSCs can be attributed to the matched work function, reduced interface resistance and smaller crystal particles of Co_20.85_Se_79.15_, which offers a much higher number of active sites for adsorption and reduction of I_3_^‒^ ions. Therefore, the reduced electron-transfer resistance between the electrolyte and the Co_20.85_Se_79.15_ CE can efficiently accelerate the reduction reaction and the electron transport, resulting in the enhanced efficiency of the DSSCs. Furthermore, a dark current, the characteristic feature associated with recombination reactions between photogenerated electrons at the conduction band of TiO_2_ and the electrolyte (I_3_^‒^), was investigated as shown in Fig. 3(b). Clearly, the Co_20.85_Se_79.15_ alloy exhibited the smallest dark current density compared to that of pristine Pt, other alloys (Ru_81.09_Co_18.91_ and Ru_80.55_Se_19.45_) and pure metals (Ru, Se and Co), demonstrating suppression of the electron-loss reaction. The result can be explained by the fact that the Co_20.85_Se_79.15_ alloy can accelerate the kinetics of the reduction reaction, namely, I_3_^‒^ + 2e^‒^ = 3I^‒^ and the regeneration rate of dye molecules. Therefore, the Co_20.85_Se_79.15_ alloy possesses advantages, such as a higher number of active sites, matched energy levels and reduced interface resistance, resulting in a fast electrolyte (I_3_^‒^) reduction at the electrolyte/CE interface instead of the recombination with electrons originated from the TiO_2_ conduction band.

**Table 2:** Photovoltaic parameters of different CEs based DSSCs under AM 1.5 G sunlight (100 mW cm^‒2^) illumination.

CEs	*J*_*sc*_ (mA cm^‒2^)	*V*_oc_ (V)	*FF*	*η* (%)
Ru_81.09_Co_18.91_	14.07	0.66	0.49	4.57
Ru_80.55_Se_19.45_	13.90	0.64	0.43	3.82
Co_20.85_Se_79.15_	15.91	0.71	0.62	7.08
Ru	13.50	0.67	0.36	3.22
Se	4.59	0.62	0.12	0.34
Co	3.43	0.62	0.13	0.27
Pt	14.72	0.69	0.57	5.80

*J*_sc_: Short-circuit current density, *V*_oc_: Open-circuit voltage, *FF*: fill factor, *η***:** Photo-electric conversion efficiency.

**Figure 4:**
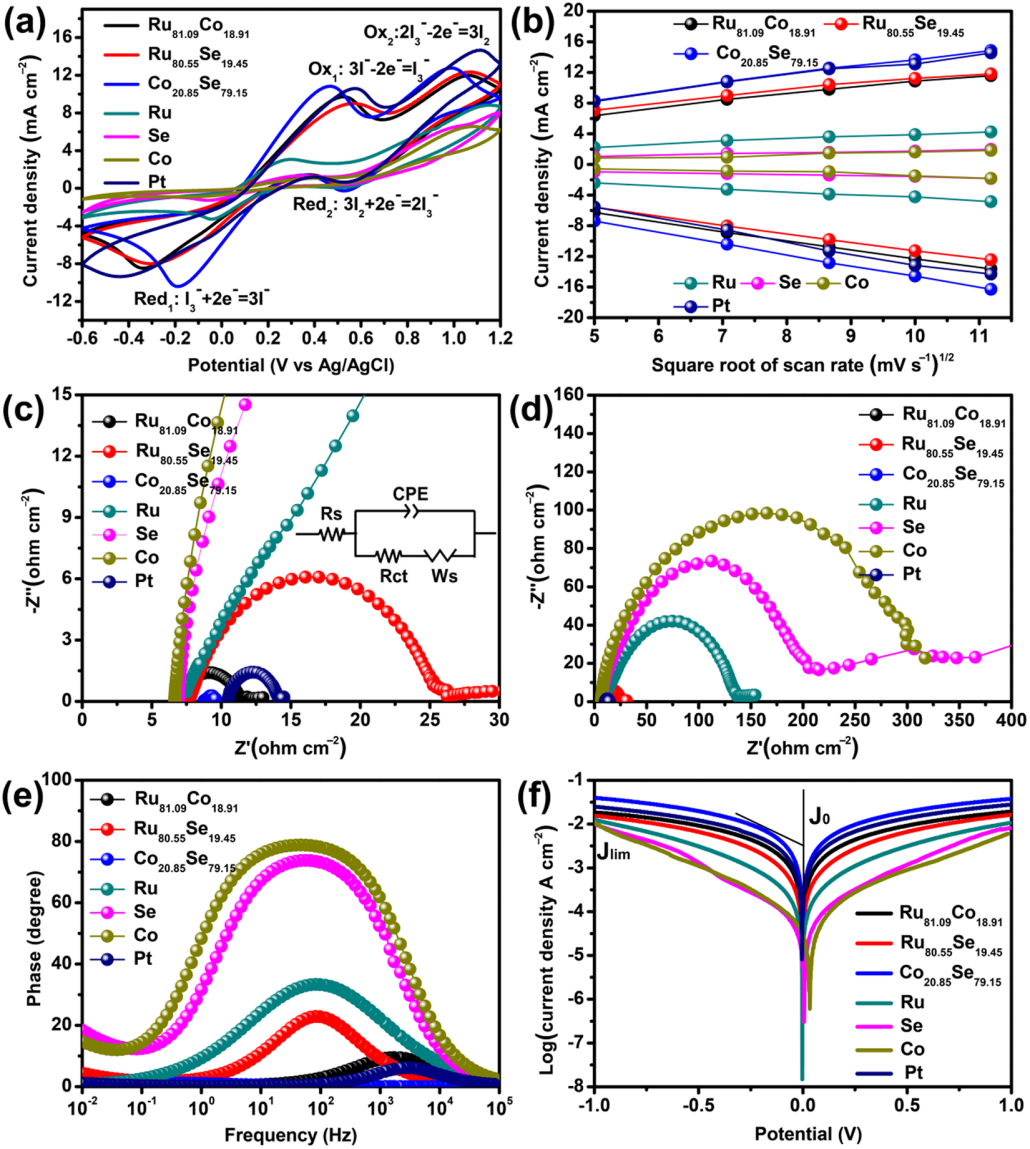
(**a**) CV plots of different CEs based cells in the N_2_ cleaned I_3_^‒/^I^‒^ redox couple electrolyte measured at a scan rate of 50 mV s^‒1^. (**b**) Relationship between square root of scan rates and peak current density. (**c-d**) Nyquist EIS curves, (**e**) Bode plots and (**f**) Tafel polarization plots of symmetric dummy devices (CE/electrolyte/CE).

**Figure 5:**
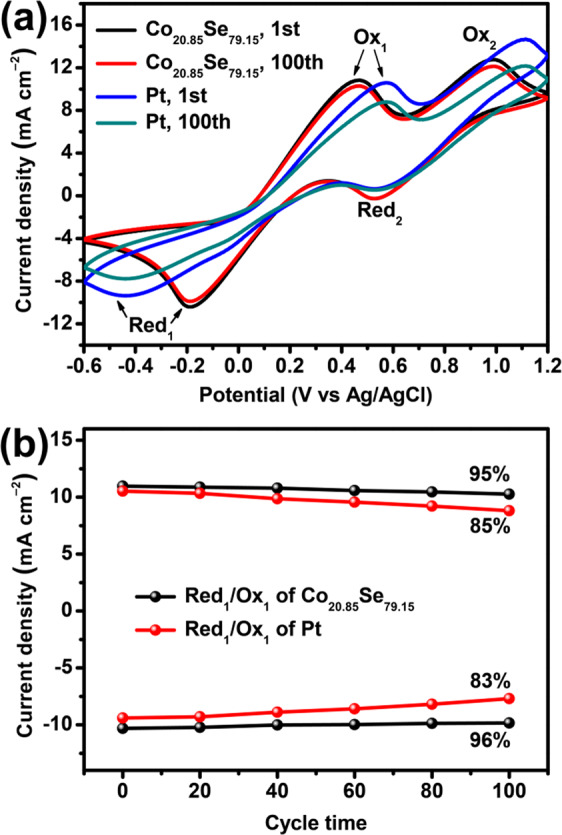
(**a**) 100 cycles of CV plots for Co_20.85_Se_79.15_ and Pt CEs at 50 mV s^‒1^ scan rate in I_3_^‒^/I^‒^ redox electrolyte, (**b**) Changes between cycle number and redox peak current density.

The electrocatalytic activities of the alloy CEs towards I_3_^‒^ reduction were also investigated by electrochemical methods as shown in Fig. 4. Figure 4(a) shows cyclic voltammogram (CV) of the different CEs where two couples of redox peaks can be seen in the CV plots. The redox peaks on the left side of the figures correspond to the I_3_^‒^ → I^‒^ process (red_1_: I_3_^‒^ + 2e^‒^ = 3I^‒^, ox_1_: 3I^‒^ - 2e^‒^ = I_3_^‒^) while the ones on the right correspond to the I_2_ → I_3_^‒^ process (red_2_: 3I_2_ + 2e^‒^ = 2I_3_^‒^, ox_2_: 2I_3_^‒^ - 2e^‒^ = 3I_2_)^26^. Since the I_3_^‒^ → I^‒^ reduction process is the dominating reaction in I_3_^‒^/I^‒^-based electrolyte systems, we mainly focus on the left redox peaks. The plot shows that the redox peaks of alloy CEs are much stronger than those of the pristine metals (Ru, Se and Co), suggesting that the alloy CEs have a superior catalytic activity toward I_3_^‒^ reduction. Table 3 shows electrochemical parameters obtained from CV plots and EIS at different CEs where the *J*_red1_ and *E*_red1_ represent the peak current density and potential of reduction reaction (red_1_), respectively. Because *J*_red1_ is a key parameter for estimating the electrocatalytic activity of CEs^27^, clearly, the *J*_red1_ decreases in the order Co_20.85_Se_79.15_ > Pt> Ru_81.09_Co_18.91_ > Ru_80.55_Se_19.45_ > Ru> Se> Co, highlighting the superior catalytic properties of the Co_20.85_Se_79.15_ alloy. Moreover, the Randles-Sevcik theory was used to investigate the ion diffusion at the electrolyte/CE interface. The electron diffusion coefficient, *D*_n_, was determined from the equation *J*_red1_ = *kn*^1.5^*AD*_n_^0.5^*C*_0_*v*^0.5^ where *C*_0_ is the I_3_^‒^/I^‒ ^ion concentration, *v* is the scan rate, *A* is the active area of the CEs, *n* is the number of electrons involved in the reduction process and *K* is a constant^28,29^. As a result, the calculated *D*_n_ values of Ru_81.09_Co_18.91_, Ru_80.55_Se_19.45_, Co_20.85_Se_79.15_, Ru, Se, Co and Pt were 2.47×10^‒5^, 2.22×10^‒5^, 3.67×10^‒5^, 0.37×10^‒5^, 0.06×10^‒5^, 0.03×10^‒5^ and 3.05×10^‒5^, respectively. The improved *D*_n_ of the Co_20.85_Se_79.15_ alloy denotes the faster diffusion kinetics of I_3_^‒^ ions between electrolyte and CEs. The higher number of active sites and the ligand effect between Co and Se are also expected to contribute the enhanced catalytic properties of the corresponding alloy CEs. Figure 4(b) shows the relationship between the square root of the scan rates and the peak current density where CV plots obtained for the CEs at different scan rates are shown in Figure S4. Obviously, the peak current density of reduction and oxidation increased almost linearly with the scan rate, indicating that the electrochemical reaction is controlled by the diffusion behavior of I_3_^‒^ ions at the electrolyte/CE interface^30^. In order to examine the internal electron transfer kinetics at the electrolyte/CE interface, Nyquist EIS curves of symmetrical devices, consisting of two identical CEs and the electrolyte, were measured and displayed in Fig. 4(c,d), respectively. The charge-transfer resistance (*R*_ct_) between electrolyte and CEs determined by fitting the Nyquist plots increase in the following order of Co_20.85_Se_79.15_ < Pt <Ru_81.09_Co_18.91_ < Ru_80.55_Se_19.45_ < Ru <Se <Co where the equivalent circuit is displayed in Fig. 3(c). A smaller *R*_ct_ denotes a rapid electron transport kinetics at the electrolyte/CE interface (Table 3). Therefore, the catalytic reactions are effectively accelerated by the Co_20.85_Se_79.15_ alloy CEs and can be further confirmed by examining the interfacial electron lifetimes (*τ*_e_ = 0.5π*f*_peak_ where *f*_peak_ represents the peak frequency in the Bode plots displayed in Fig. 4e) as shown in Fig. 4(e) and Table 4 shows electrochemical parameters obtained from the EIS and Tafel polarization plots based on CE/electrolyte/CE structures^31^. The calculated lifetimes varied in the following order of Co_20.85_Se_79.15_ (15.91 μs) <Pt (59.28 μs) <Ru_81.09_Co_18.91_ (93.18 μs) <Ru_80.55_Se_19.45_ (518.10 μs) <Ru (2334.83 μs) <Se (2833.37 μs) <Co (3431.80 μs), respectively. Lower *τ*_e_ values indicate the faster reduction kinetics of I_3_^‒^ ions, yielding the improved electrocatalytic ability of the CEs. These results are in good agreement with the CV plots. Based on the Tafel polarization plots in Fig. 4(f), the exchange and diffusion-limited current densities (*J*_0_ and *J*_lim_, respectively) can be calculated from the equations^9^ *J*_0_ = *RT*/*nFR*_ct_ and *J*_lim_ = 2*nFCD*_n_/*l*, respectively (Table 4). The obtained *J*_0_ and *J*_lim_ values also decreased in the order Co_20.85_Se_79.15_ > Pt> Ru_81.09_Co_18.91_ > Ru_80.55_Se_19.45_ > Ru> Se> Co, in good agreement with the CV and EIS results. Therefore, the present results allow us to conclude that binary Co_20.85_Se_79.15_ alloy CEs possess superior catalytic abilities compared with alloy and pure Pt CEs. Furthermore, 100 cycles of CV plots from Co_20.85_Se_79.15_ and Pt CE in I_3_^‒^/I^‒^ redox electrolyte were used to evaluate the electrochemical stability as shown in Fig. 5, for which the peak current density *J*_red1_ of Co_20.85_Se_79.15_ CE can retain 96% of its initial value while the *J*_red1_ of Pt CE decreases to 83% of initial value. The results indicate that the alloy Co_20.85_Se_79.15_ CE possesses superior electrochemical stability in the I_3_^‒^/I^‒^ supported electrolyte.

**Table 3:** Electrochemical parameters obtained from CV plots and EIS based on CE/electrolyte/CE devices of different CEs.

CEs	*J*_*red1*_ (mA cm^‒2^)	*E*_*red1*_ (V)	*D*_*n*_ (cm^2^ s^‒1^)	*R*_ct_ (Ω cm^‒2^)
Ru_81.09_Co_18.91_	‒8.475	‒0.338	2.47**×**10^‒5^	4.32
Ru_80.55_Se_19.45_	‒8.033	‒0.292	2.22**×**10^‒5^	18.27
Co_20.85_Se_79.15_	‒10.320	‒0.191	3.67**×**10^‒5^	1.26
Ru	‒3.291	‒0.041	0.37**×**10^‒5^	124.86
Se	‒1.267	‒0.059	0.06**×**10^‒5^	198.80
Co	‒0.873	‒0.130	0.03**×**10^‒5^	291.46
Pt	‒9.406	‒0.425	3.05**×**10^‒5^	3.45

**Table 4:** Electrochemical parameters obtained from the EIS and Tafel polarization plots based on CE/electrolyte/CE structures.

CEs	Bode parameters f_peak_ (Hz)	τ_e_ (μs)	Tafel parameters J_0_ (mA cm^‒2^)	J_lim_ (mA cm^‒2^)
Ru_81.09_Co_18.91_	1709	93.18	2.97	20.89
Ru_80.55_Se_19.45_	82.5	518.10	0.70	19.50
Co_20.85_Se_79.15_	10010	15.91	10.19	31.63
Ru	68.2	2334.83	0.36	12.60
Se	56.2	2833.37	0.28	10.23
Co	46.4	3431.80	0.26	10.96
Pt	2686	59.28	3.72	25.12

**Figure 6:**
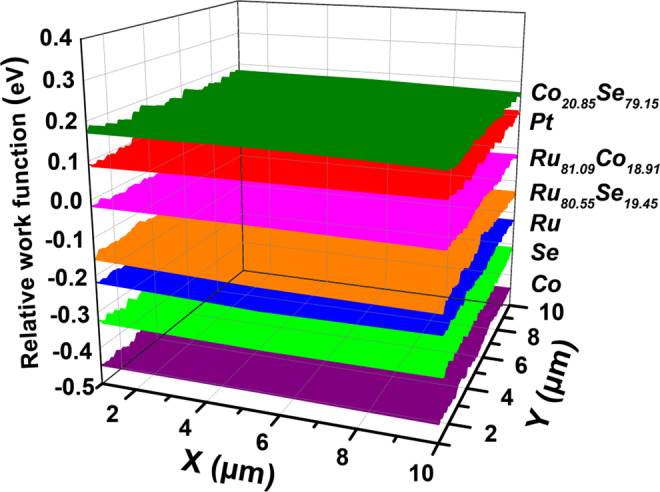
Diagrams of relative work function for various alloy and pristine Pt CEs.

In order to further investigate the internal mechanism of the CEs, the work functions of the different CEs were determined by SKPM using a gold probe (5.1 eV) as a standard reference, and are shown in Fig. 6. According to the SKPM analysis, the work functions of Ru_81.09_Co_18.91_, Ru_80.55_Se_19.45_, Co_20.85_Se_79.15_, Ru, Se and Co are ‒5.12, ‒5.25, ‒4.94, ‒5.33, ‒5.40 and ‒5.55 eV whereas the corresponding value for the pure Pt electrode is ‒5.01 eV, respectively. Therefore, the work functions of the Co_20.85_Se_79.15_ alloy CE show a better match with the potential (‒4.90 eV) of the I_3_^‒^/I^‒^ redox electrolyte^18^ compared with those of the other CEs, thus resulting in improved electrocatalytic performance. The good match of the Co_20.85_Se_79.15_ CE work function can be attributed to the ligand effect of the Co and Se transition metals, which would reduce the bond energy between atoms and free electrons^32^. As a result, the electronic configurations of Co and Se atoms near the surface are readjusted in such a way that electrons are prone to participate in the electrolyte (I_3_^‒^) reduction process. Furthermore, the charge transport resistance is defined as the difference between the work function of the CE and the potential of the I_3_^‒^/I^‒^ redox electrolyte of DSSCs^11,33^. For this reason, a lower energy drop would efficiently accelerate the electron transport from electrocatalyst CEs to I_3_^‒^^11,33^, which is in good agreement with the above EIS analysis. In this case, the superior electrocatalytic activity of the Co_20.85_Se_79.15_ CE toward the I_3_^‒^ electrolyte can definitely hinder the recombination reaction between I_3_^‒^ and excited electrons at the conduction band of nanocrystalline TiO_2_, thereby creating an increased photogenerated current density.

**Figure 7:**
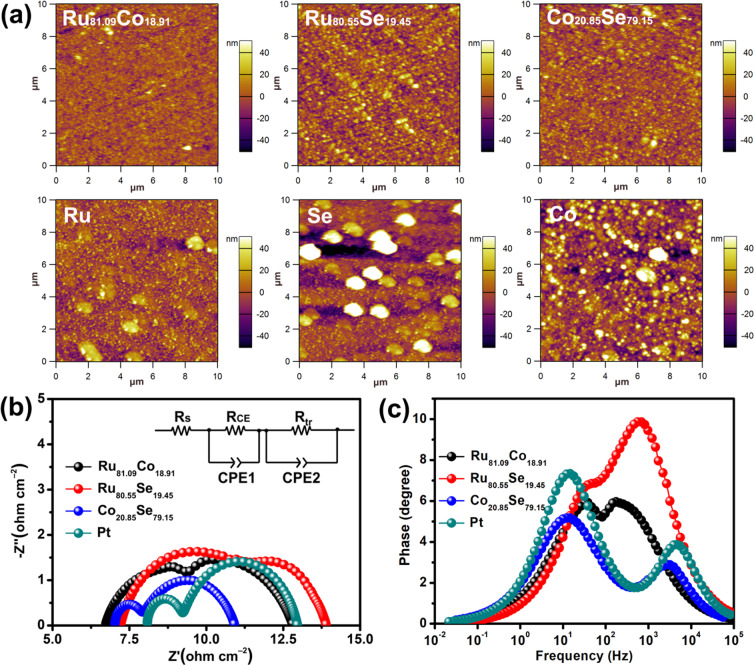
(**a**) Scanning kelvin probe images of different CEs, (**b**) Nyquist EIS plots of the DSSCs with various alloy CEs, and (**c**) Bode phase plots (under sunlight conditions).

Scanning Kelvin Probe Microstructures are also employed to characterize the surface nanostructures of various CEs as shown in Fig. 7(a). Relatively uniform distribution of Co_20.85_Se_79.15_ alloy CEs was observed, which is in agreement with the SEM. To verify the effect of alloy CEs on the internal transfer kinetics of DSSCs, the Nyquist and Bode phase curves (under light illumination) of DSSCs based on different CEs are displayed in Fig. 7(b,c) and the inset in Fig. 7(b) shows the equivalent circuit where *R*_tr_ and *R*_CE_ represent the charge-transfer resistances at the dye-sensitized TiO_2_/electrolyte interface and electrolyte/CE interfaces for DSSCs, respectively. The electrochemical parameters obtained by fitting the impedance spectra are summarized in Table 5. The DSSC based on the Co_20.85_Se_79.15_ alloy CE exhibited a lower *R*_tr_ (3.06 Ω cm^‒2^), suggesting a fast I_3_^‒^ to I^‒^ reduction kinetics at the electrolyte/CE interface, where the rapid accumulation of I^‒^ ions can then facilitate their diffusion to the dye-sensitized TiO_2_ photoanode/electrolyte interface. Furthermore, the photogenerated electrons in the nanocrystalline TiO_2_ photoanode of the Co_20.85_Se_79.15_ alloy DSSC showed a longer *τ*_e_ value compared with those of other devices (Table 5). This result demonstrates that dye molecules are rapidly regenerated by I^‒^ ions, thus enabling fast transport of photogenerated electrons in the mesoporous TiO_2_ nanocrystal photoanode, resulting in the superior catalytic activity of the Co_20.85_Se_79.15_ alloy CE. Furthermore, photovoltaic parameters and synthetic technology comparisons of the various Pt-free transition metal selenides CEs based DSSCs^34–39^ were provided as shown in Table 6.

**Table 5:** Electrochemical parameters obtained from the impedance spectra of various CEs based DSSCs under AM 1.5 G simulated sunlight.

Light	*R*_CE_ (Ω cm^‒2^)	*R*_tr_ (Ω cm^‒2^)	*f*_peak_ (Hz)	*τ*_e_ (ms)
Ru_81.09_Co_18.91_	3.99	3.95	38.31	4.16
Ru_80.88_Se_19.45_	5.36	4.07	46.42	3.43
Co_20.85_Se_79.15_	0.84	3.06	12.12	13.14
Pt	1.12	3.78	14.68	10.85

**Table 6:** Photovoltaic parameters comparisons of the reported Pt-free transition metal selenides counter electrode based-DSSCs.

CE materials	Method	*J*_sc_ (mA cm^‒2^)	*V*_oc_ (V)	FF	PCE (%)	PCE Pt (%)	Ref.
Co_0.85_Se	Low-t-hydrothermal	16.74	0.742	0.67	8.30	6.18	^7^
Ni_0.85_Se	Low-t-hydrothermal	16.67	0.740	0.64	7.85	6.18	^7^
Cu_0.50_Se	Low-t-hydrothermal	14.55	0.713	0.62	6.43	6.18	^7^
Ru_0.33_Se	Low-t-hydrothermal	18.93	0.715	0.68	9.22	6.18	^7^
FeSe	Low-t-hydrothermal	17.10	0.733	0.61	7.64	6.18	^7^
CoSe_2_/N-C@CC	Solution method + selenization	16.39	0.730	0.70	8.40	8.09	^20^
CoSe_2_/Porous CS	Carbonization + selenization	15.88	0.690	0.69	7.56	7.40	^21^
Co_0.85_Se	Low-t-hydrothermal	16.98	0.738	0.75	9.40	8.64	^22^
Ni_0.85_Se	Low-t-hydrothermal	15.63	0.739	0.72	8.32	8.64	^22^
NiSe-Ni_3_Se_2_	Solothermal method	16.31	0.750	0.64	7.83	7.28	^25^
NiSe_2_	Low-t-hydrothermal	15.72	0.660	0.31	3.28	6.11	^34^
Ni_0.9_Co_0.1_Se	Low-t-hydrothermal	13.46	0.690	0.39	3.68	6.11	^34^
Ni_0.5_Co_0.5_Se	Low-t-hydrothermal	15.65	0.700	0.54	6.02	6.11	^34^
CoSe_2_	Low-t-hydrothermal	14.59	0.710	0.55	5.78	6.11	^34^
CoSe_2_ NRs	High-t-hydrothermal	18.55	0.753	0.73	10.20	8.17	^35^
Co_0.5_Se	Low-t-hydrothermal	13.27	0.721	0.67	6.47	6.18	^36^
Co_0.7_Se	Low-t-hydrothermal	15.36	0.739	0.66	7.46	6.18	^36^
Co_0.85_Se	Low-t-hydrothermal	16.80	0.742	0.67	8.30	6.18	^36^
CoSe	Low-t-hydrothermal	15.47	0.743	0.67	7.75	6.18	^36^
Co_1.2_Se	Low-t-hydrothermal	11.45	0.723	0.65	5.37	6.18	^36^
Fe_0.6_Se	Low-t-hydrothermal	13.95	0.705	0.62	6.08	/	^37^
Fe_0.8_Se	Low-t-hydrothermal	16.75	0.713	0.58	6.87	/	^37^
FeSe	Low-t-hydrothermal	17.72	0.717	0.72	9.16	/	^37^
Fe_1.2_Se	Low-t-hydrothermal	14.56	0.683	0.58	5.77	/	^37^
NbSe_2_ NSs/C	Chemical + heat treating	15.58	0.770	0.65	7.80	7.90	^38^
Ni_0.33_Co_0.67_Se microsphere	Two-step hydrothermal	17.29	0.789	0.67	9.01	8.30	^39^
Co_20.85_Se_79.15_ nanoparticles	Electrodeposition	15.91	0.710	0.62	7.08	5.80	This work

## Conclusion

In summary, tunable and Pt-free CEs based on binary alloys (Ru_81.09_Co_18.91_, Ru_80.55_Se_19.45_, and Co_20.85_Se_79.15_) have been synthesized by a simple electrodeposition approach. The results indicate that Co_20.85_Se_79.15_ alloy CEs possess outstanding electrocatalytic properties towards I_3_^‒^ reduction, which can be attributed to their higher number of active sites, reduced interfacial resistance, and matched work function with the I_3_^‒^/I^‒^ redox electrolyte. The Co_20.85_Se_79.15_ alloy-based CE device displays a higher PCE of 7.08% compared with that of a pure Pt CE (5.80%) as well as preferable stability. Although the obtained alloy composition and performance could be further optimized, the easy synthesis method and hopeful efficiency indicate that electrochemical technologies have significant potential for the development of low-cost, high efficiency and stable DSSCs.

## Experimental Section

### Synthesis of RuCo/RuSe/CoSe alloy and pristine Pt CEs

The RuCo, RuSe and CoSe alloys were synthesized by electrochemical co-electrodeposition on a cleaned fluorine-doped tin oxide (FTO, sheet resistance 12 Ω sq^‒1^, purchased from Sunlaite) glass substrate, using a galvanostatic approach on the electrochemical workstation. First, a solution (A: 3 mM RuCl_3_, 2 mM CoSO_4_; B: 3 mM RuCl_3_, 2 mM SeO_2_; C: 2 mM CoSO_4_, 3 mM SeO_2_) and 100 mM LiCl were dispersed by ultrasonic waves for 30 min. Then, the deposition was carried out in a three-electrode system equipped with an FTO substrate (working electrode), a Pt electrode (counter electrode) and Ag/AgCl (reference electrode). The procedure was performed at a current density of 0.25 mA cm^‒2^ for 600 s. Finally, the synthesized alloy CEs were rinsed with deionized water and dried at 80 °C in a vacuum furnace. For comparison, pristine metal CEs were also prepared with a 5 mM RuCl_3_, 5 mM CoSO_4_, 5 mM SeO_2_ under the same conditions, respectively. The pristine Pt CE was also prepared by cyclic voltammetry in the range of ‒0.8~0.6 V by using 5 mM H_2_PtCl_6_ solution, the scan parameter was controlled at 10 mV s^‒1^ for 5 cycles.

### Electrochemical characterization

A CHI760E electrochemical workstation equipped with a three-electrode device was utilized to assess the electrocatalytic performance of the prepared CEs. Cyclic voltammetry (CV) measurements were carried out in an auxiliary electrolyte consisting of 500 mM LiClO_4_, 10 mM I_2_ and 50 mM LiI in acetonitrile at scan rates of 25, 50, 75, 100 and 125 mV s^‒1^, respectively. Electrochemical impedance spectroscopy (EIS) measurements were performed on symmetrical dummy solar cells with identical CE structures at frequencies ranging from 0.01 to 10^5^ Hz and amplitude of 10 mV in air, respectively. Tafel polarization plots were also recorded on symmetrical cells at a scan rate of 10 mV s^‒1^. Then, EIS measurements of the DSSCs were performed with an amplitude of 10 mV, under sunlight illumination.

### Fabrication of DSSCs

A TiO_2_ nanoparticle film-based on the FTO glass substrate was prepared according to the procedure described in our previous work. Specific as follows: a mixed solution of 20 mL ethanol and 8 mL tetrabutyl titanate was magnetically stirred for 30 min. Then, the acquired solution was added to a solution consisting of 5 mL deionized water and 40 mL acetic acid with magnetic stirring for 2 h. Afterward, the solution underwent a hydrothermal process at 230 °C for 12 h. Finally, the photoanode was obtained after washing the products and spin-coating TiO_2_ nanoparticle on FTO glass. The thickness and active area of the photoanode film were controlled to 10 μm and 0.25 cm^2^, respectively. Then, the prepared TiO_2_ photoanode was sensitized in a 0.50 mM N719 ethanol solution for 18 h. Afterward, the DSSCs were obtained by assembling the dye-sensitized TiO_2_ photoanode and CEs with Surlyn tape, followed by injection of the I_3_^‒^/I^‒^ redox electrolyte (0.05 M I_2_, 0.1 M LiI, 0.3 M 1,2-dimethyl-3-propylimidazoliumiodide, and 0.5 M tertbutylpyridine).

### Photoelectrochemical measurements

The photovoltaic performances of the assembled DSSCs were characterized using a CHI760E electrochemical workstation under simulated solar irradiation with an intensity of 100 mW cm^‒2^ (using a 500 W CHF-XM500 xenon arc lamp as light source). A standard Si solar cell was used to calibrate the intensity of the solar simulator. EIS measurements of the devices were also conducted at frequency ranges of 0.01–10^5^ Hz, with a bias voltage and a AC amplitude of 0.68 V and 10 mV, respectively. Results were analyzed by the Z-view software using the corresponding equivalent circuit.

### Characterization

The phase structures of the synthesized alloy CEs and pristine metals were characterized by powder X-ray diffraction (XRD, Bruker D8 Advance) with Cu K_α_ radiation (*λ* ~ 0.154 nm) and X-ray photoelectron spectroscopy (XPS, Thermo Fisher Scientific). Field-emission scanning electron microscopy (FESEM, JEOL JSM-6701F, Japan) and energy-dispersive spectroscopy (EDS) measurements were carried out to inspect the microstructures and elemental compositions of the alloy CEs. Scanning Kelvin probe microscopy (SKPM, CH020) was used to determine the relative work functions of the different CEs, with a gold probe serving as a reference electrode (5.1 eV).

## Supplementary information

Supplementary Information.
