# Supplementary material for: Interface Engineered Binary Platinum Free Alloy-based Counter Electrodes with Improved Performance in Dye-Sensitized Solar Cells
Source: Sci Rep. 2020 Jun 8;10:9157. doi: 10.1038/s41598-020-64965-7 (PMC7280534; doi:10.1038/s41598-020-64965-7)
Supplement: Supplementary file 1 — Supplementary Information. [file 41598_2020_64965_MOESM1_ESM.docx]

**Supplementary information**

**Interface Engineered B****inary Platinum Free Alloy-based Counter Electrodes with Improved Performance in Dye-Sensitized Solar Cells**

Wen-Wu Liu^1,2^,* Wei Jiang^1,2^, Yu-Cheng Liu^1,2^, Wen-Jun Niu^1,2^, Mao-Cheng Liu^1,2^, Ling-Bin Kong^1,2^, Ling Lee^3, 6^, Zhiming M. Wang^6^*,* Yu-Lun Chueh^3, 4, 5^*

^1^State Key Laboratory of Advanced Processing and Recycling of Nonferrous Metals, Lanzhou University of Technology, Lanzhou 730050, PR China

^2^College of Materials Science and Engineering, Lanzhou University of Technology, Lanzhou 730050, PR China

*E-mail: lww06080428@163.com

^3^Department of Materials Science and Engineering, National Tsing Hua University, Hsinchu 30013, Taiwan.

^4^Frontier Research Center on Fundamental and Applied Sciences of Matters, National Tsing Hua University, Hsinchu 30013, Taiwan.

^5^Department of Physics, National Sun Yat-Sen University, Kaohsiung, 80424, Taiwan.

^6^Institute of Fundamental and Frontier Sciences, University of Electronic Science and Technology of China, Chengdu, P. R. China

*E-mail: ylchueh@mx.nthu.edu.tw


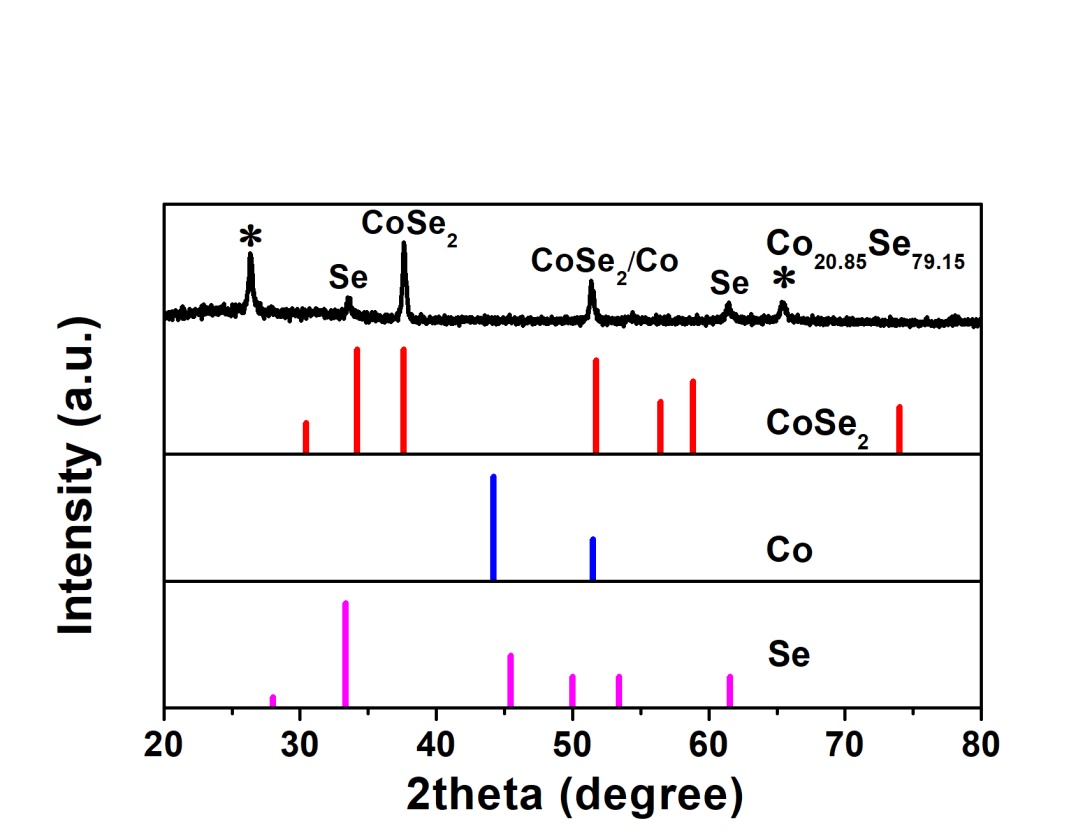


**Figure S1.** XRD patterns of the different alloys and pristine metal CEs, asterisk (*) represent FTO glass.


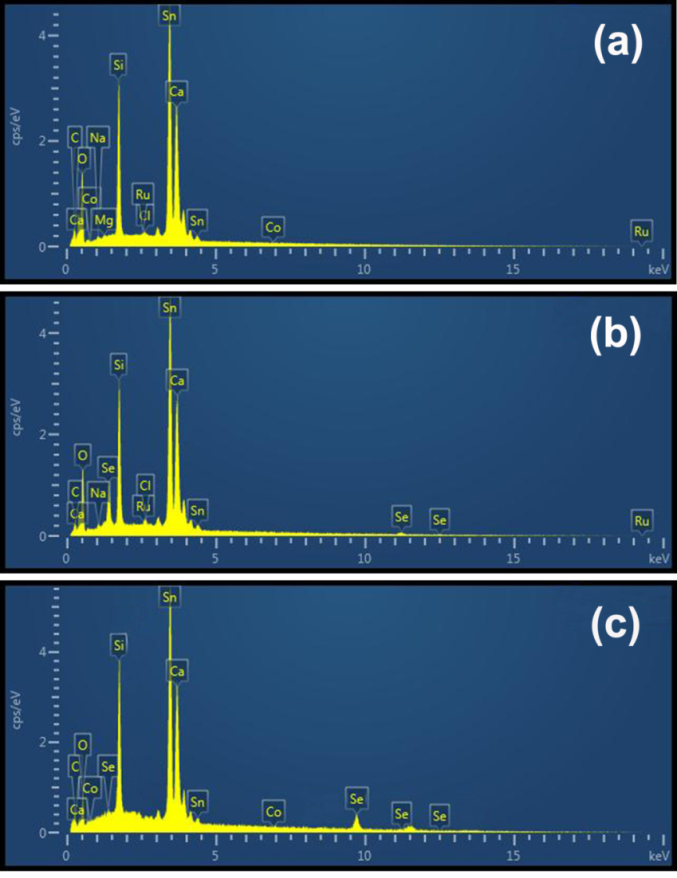


**Figure S2.** EDS patterns of the different alloy CEs (a) Ru_81.09_Co_18.91_, (b) Ru_80.55_Se_19.45_, (c) Co_20.85_Se_79.15_


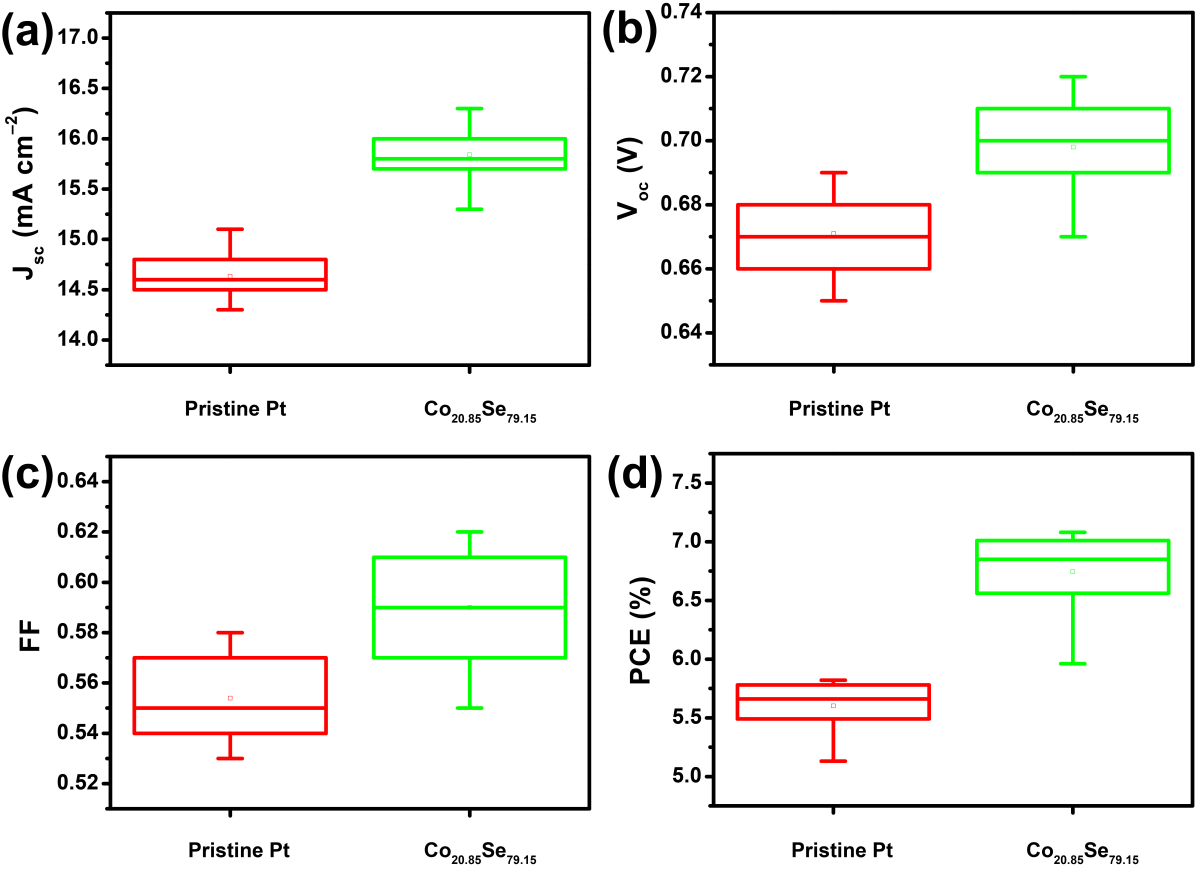


**Figure S3.** Box-charts of the parameters (a) *J*_sc_, (b) *V*_oc_, (c) FF and (d) PCE of 10 cells based on pristine Pt and Co_20.85_Se_79.15_, respectively.


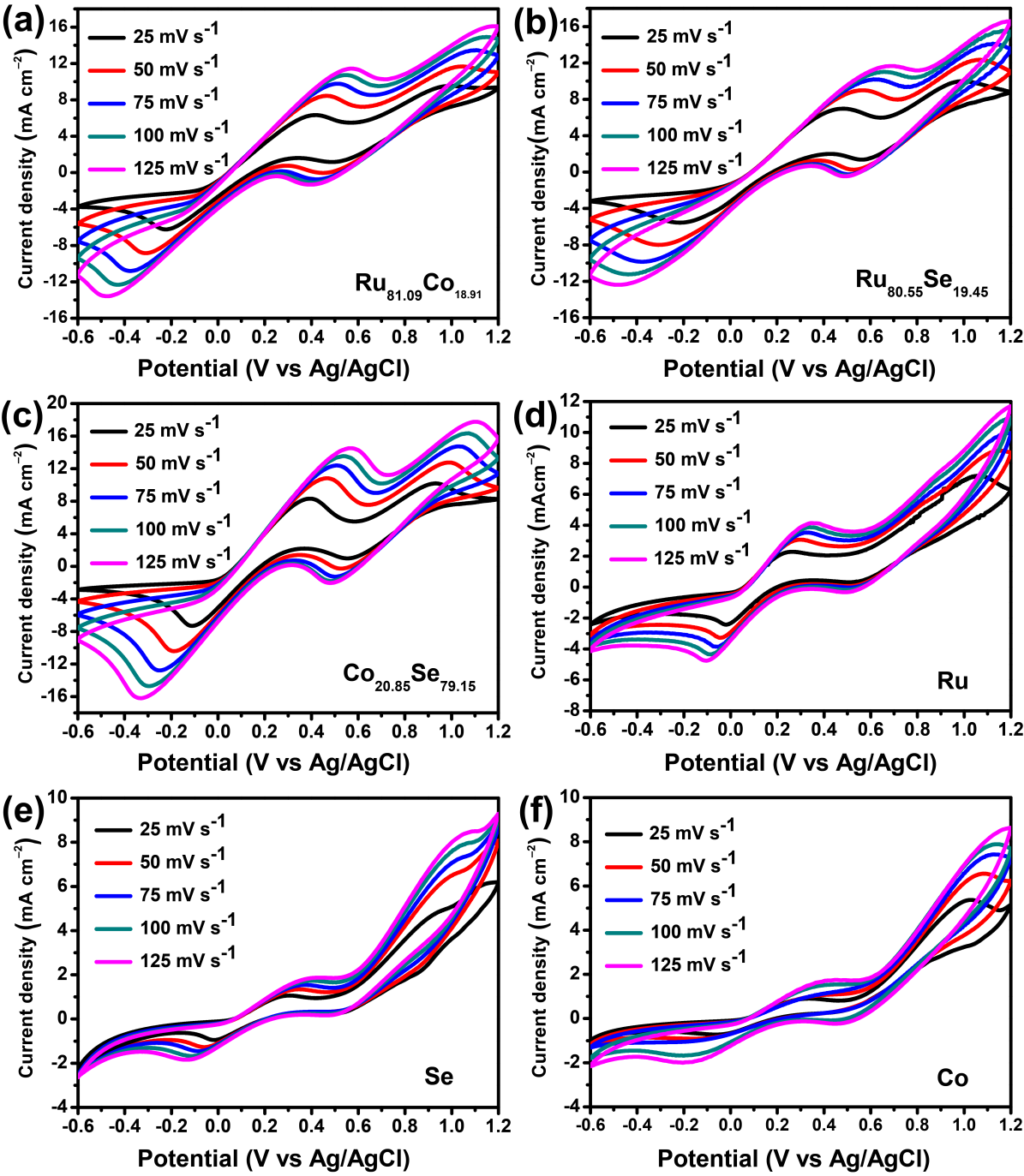


**Figure S4.** The CV plots of different CEs recorded at different scan rates from 25 to 125 mV s^-1^. (a) Ru_81.09_Co_18.91_, (b) Ru_80.55_Se_19.45_, (c) Co_20.85_Se_79.15_, (d) Ru, (e) Se, (f) Co.
